# New Mammalian Glycerol-3-Phosphate Phosphatase: Role in β-Cell, Liver and Adipocyte Metabolism

**DOI:** 10.3389/fendo.2021.706607

**Published:** 2021-07-13

**Authors:** Elite Possik, Anfal Al-Mass, Marie-Line Peyot, Rasheed Ahmad, Fahd Al-Mulla, S. R. Murthy Madiraju, Marc Prentki

**Affiliations:** ^1^ Departments of Nutrition, Biochemistry and Molecular Medicine, and Montreal Diabetes Research Center, CRCHUM, Montréal, QC, Canada; ^2^ Department of Medicine, McGill University, Montréal, QC, Canada; ^3^ Immunology & Microbiology Department, Dasman Diabetes Institute, Dasman, Kuwait

**Keywords:** glycerol-3-phosphate phosphatase, phosphoglycolate phosphatase, glycerolipid/free fatty acid cycle, insulin secretion, nutri-stress, cardiometabolic diseases, type 2 diabetes, obesity

## Abstract

Cardiometabolic diseases, including type 2 diabetes, obesity and non-alcoholic fatty liver disease, have enormous impact on modern societies worldwide. Excess nutritional burden and nutri-stress together with sedentary lifestyles lead to these diseases. Deranged glucose, fat, and energy metabolism is at the center of nutri-stress, and glycolysis-derived glycerol-3-phosphate (Gro3P) is at the crossroads of these metabolic pathways. Cellular levels of Gro3P can be controlled by its synthesis, utilization or hydrolysis. The belief that mammalian cells do not possess an enzyme that hydrolyzes Gro3P, as in lower organisms and plants, is challenged by our recent work showing the presence of a Gro3P phosphatase (G3PP) in mammalian cells. A previously described phosphoglycolate phosphatase (PGP) in mammalian cells, with no established physiological function, has been shown to actually function as G3PP, under physiological conditions, particularly at elevated glucose levels. In the present review, we summarize evidence that supports the view that G3PP plays an important role in the regulation of gluconeogenesis and fat storage in hepatocytes, glucose stimulated insulin secretion and nutri-stress in β-cells, and lipogenesis in adipocytes. We provide a balanced perspective on the pathophysiological significance of G3PP in mammals with specific reference to cardiometabolic diseases.

## Introduction

Metabolism of macronutrients including carbohydrates, amino acids and fats converges on the generation of a three-carbon moiety, glycerol, either in the free form or as glycerol-3-phosphate (Gro3P), which forms the backbone of glycerolipids in almost all the species. Glycerolipids, including triglycerides and phospholipids make up a large part of the fat in our body, either as depot fat or as membrane components (1). The glycerol moiety of these glycerolipids is derived from glucose metabolism, dietary fat and *via* glyceroneogenesis, particularly under fasting and high sucrose diet conditions (2–5). It is generally believed that free glycerol is produced and released from cells mainly during the hydrolysis of glycerolipids (6) in higher animals, including humans (4, 5). Although some early studies indicated the likely presence of a specific enzyme in animal cells that can generate glycerol directly from the hydrolysis of Gro3P, such enzyme was known to be present only in plants and lower organisms (7–9).

The present review focuses on the identification of a specific Gro3P phosphatase (G3PP) in mammalian cells and its most plausible physiological function, specifically addressing its role in controlling glucose, lipid and energy metabolism. We also present a balanced view on the physiological relevance of the various suggested substrates of this enzyme, with a discussion on the importance of G3PP in preventing glucotoxicity/nutri-stress and the control of glucose stimulated insulin secretion (GSIS) in β-cells, in the regulation of lipogenesis in liver and adipose tissue, and in slowing down hepatic glucose production. Finally, we address the regulation of G3PP and its role in cardiometabolic diseases.

## Early Evidence for the Presence of Gro3P Phosphatase in Animal Cells

Even though lipolysis is considered to be the main source of free glycerol in mammalian cells, few earlier studies suggested that glycerol may be formed *via* non-lipolytic pathways during glycolysis. Thus, as much as 15 to 20% of plasma glycerol was thought to be derived from non-adipose tissue sources including perirenal fat or skeletal muscle lipolysis or possibly by the hydrolysis of Gro3P in long-term fasting human subjects, on the basis of stable isotope labeling (10) and in rats and monkeys (11), but no specific enzyme was described for this process. Similarly, it was noticed that significant levels of glycerol are derived directly from glucose in ischemic rat brain (12) and ischemic cardiac tissue (13). In addition, in the fish *Osmerus mordax* (Rainbow smelt) high concentrations of glycerol are generated as a cryoprotective mechanism directly from glucose, glycogen and amino acids and not from lipolysis, probably involving a glycerol-3-phosphatase, even though no specific enzyme was identified (14, 15).

Lipolysis, measured as glycerol release, was implicated in the regulation of GSIS by pancreatic β-cells (16, 17), and it was assumed that glycerolipid hydrolysis is the only source of free glycerol in mammalian cells (4, 5). However, despite the loss of adipose triglyceride lipase (ATGL), which catalyzes the first step of triglyceride hydrolysis (18, 19), glycerol is still produced in significant quantities in the pancreatic islets from whole-body ATGL-KO (20) and β-cell specifc ATGL-KO (21) mice, at high concentrations of glucose, suggesting a non-lipolytic origin of glycerol. We reported that orlistat, a powerful pan-lipase inhibitor, totally inhibits GSIS as well as lipolysis, measured as free fatty acid (FFA) release, in pancreatic β-cells (22), but not glycerol release at elevated glucose concentrations (>10 mM), suggesting that pancreatic β-cells can produce glycerol from glucose, *via* non-lipolytic pathways (22). This observation led us to conduct a thorough search using BLAST analysis for mammalian proteins that are homologous to the known microbial (yeast and bacteria) glycerol-3-phosphate phosphatase enzymes (8, 23), and to the identification of previously described phosphoglycolate phosphatase (PGP), as the potential mammalian G3PP (22).

PGP is an evolutionarily conserved enzyme that can hydrolyze various phospho-metabolites, under normal physiological as well as stress conditions (24, 25). In lower organisms and plants, PGP and G3PP are products of separate genes and hydrolyze 2-phosphoglycolate and Gro3P, respectively. However, in mammalian cells, a single gene (*PGP*) product, i.e., G3PP, appears to catalyze the hydrolysis of Gro3P under normal physiological conditions, and also 2-phosphoglycolate and other phospho-substrates produced in stress conditions, as reviewed here.

## Mammalian PGP: Names, Substrate Specificity and Enzyme Kinetics

In mammals, *PGP* gene product has been described with different names and ascribed different functions. This protein was first described in human red blood cells (RBC) as phosphoglycolate phosphatase, based on the sequence similarity to the plant and bacterial PGP enzymes, with similar substrate specificity, i.e., high activity towards 2-phosphoglycolate (26, 27). Later, this protein was called as aspartate-based, ubiquitous, Mg^2+^-dependent phosphatase (AUM), showing hydrolytic activity with phosphotyrosine containing peptides, with implications in the modulation of epidermal growth factor receptor (EGFR) signaling (25, 28–30). However, considering the extremely low cellular levels of 2-phosphoglycolate under physiological conditions and as the catalytic efficiency of purified PGP towards the phosphotyrosine peptides was ∼1,000-fold less than that of classical tyrosine phosphatases like PTP1B, TCPTP, or SHP1, the proposed physiological role of mammalian PGP in the hydrolysis of either 2-phosphoglycolate or phosphotyrosine residues is questionable.

The more likely function of RBC PGP was suggested to be the hydrolysis of 2-phospholactate, formed as a by-product of pyruvate kinase (31). More recently, PGP was shown to dephosphorylate 4-phosphoerythronate and 2-phospholactate, toxic by-products of glyceraldehyde-3-phosphate dehydrogenase (GAPDH) and pyruvate kinase, respectively. The kinetic parameters and the available intracellular substrate concentrations likely provide a clue about the more appropriate physiological substrate of G3PP in mammalian cells. The reported Km of the purified mouse PGP/G3PP enzyme for 2-phosphoglycolate is 766µM; for 4-phosphoerythronate it is 247µM and for 2-phospholactate it is 174 µM (32). However, it was reported by Collard et al. (32), that in wildtype HCT116 cells 4-phosphoerythronate and also 2-phosphoglycolate are at below detection limits (<2-3 µM). Even if one assumes that normally expressed PGP/G3PP is maintaining the concentration of these metabolites at low level, the enzyme should be able to act on them. This is kinetically not favourable for PGP/G3PP expressed at normal level as these metabolites are present at nearly 100-300 fold lower concentration in wildtype cells than their respective Km values for PGP/G3PP. In addition, the catalytic rate of GAPDH to produce 4-phosphoerythronate is ~3500 fold lower than its normal function and similarly the formation of 2-phospholactate by pyruvate kinase is several orders of magnitude lower (32). Therefore, the possibility that 2-phosphoglycolate, 4-phosphoerythronate and 2-phospholactate serve as ‘physiological’ substrates for PGP/G3PP is remote, unlike glycerol-3-phosphate, which is present at above its Km concentration in cells normally, and is readily available as substrate for G3PP. However, as 4-phosphoerythronate and 2-phospholactate accumulate when PGP expression is suppressed (32–34), the possibility that PGP/G3PP may act on these substrates with very low efficiency due to their extremely low intracellular concentrations, cannot be discounted. Even though purified recombinant mouse PGP was found to show high catalytic efficiency with 2-phosphoglycolate, 4-phosphoerythronate, and 2-phospholactate, and relatively lower activity with Gro3P (22, 32), it also shows very high activity with the non-physiological substrate p-nitrophenol phosphate (30). Studies from our laboratory demonstrated that PGP actually functions as a G3PPin many cell types, hydrolysing Gro3P, normally produced in all the cells and available in sufficient concentrations (1 to 5 mM; Km, ~1 mM) to serve as a substrate for this enzyme (22, 24). Hence, the name G3PP is more appropriate for the *PGP* gene product, and is now accepted by most protein databases (Uniprot, NCBI Protein, PDB, etc.). Thus, the observed catalytic efficiencies of purified G3PP/PGP *in vitro* may not have much relevance physiologically, as it is the availability of substrate that dictates the activity of a given enzyme. Therefore, on the basis of available evidence, we suggest that the protein encoded by *PGP* in mammalian cells is poised to act on Gro3P, as its normal physiological function, but may assume a detoxification role to hydrolyze toxic phospho-metabolites, such as 2-phosphoglycolate, 4-phosphoerythronate or 2-phospholactate, which may buildup in the cells under stress conditions (22, 24, 25, 32). Additional studies, particularly *in vivo*, are needed to ascertain this possibility.

## Control of Glucose, Lipid and Energy Metabolism by G3PP/PGP

**Figure 1:**
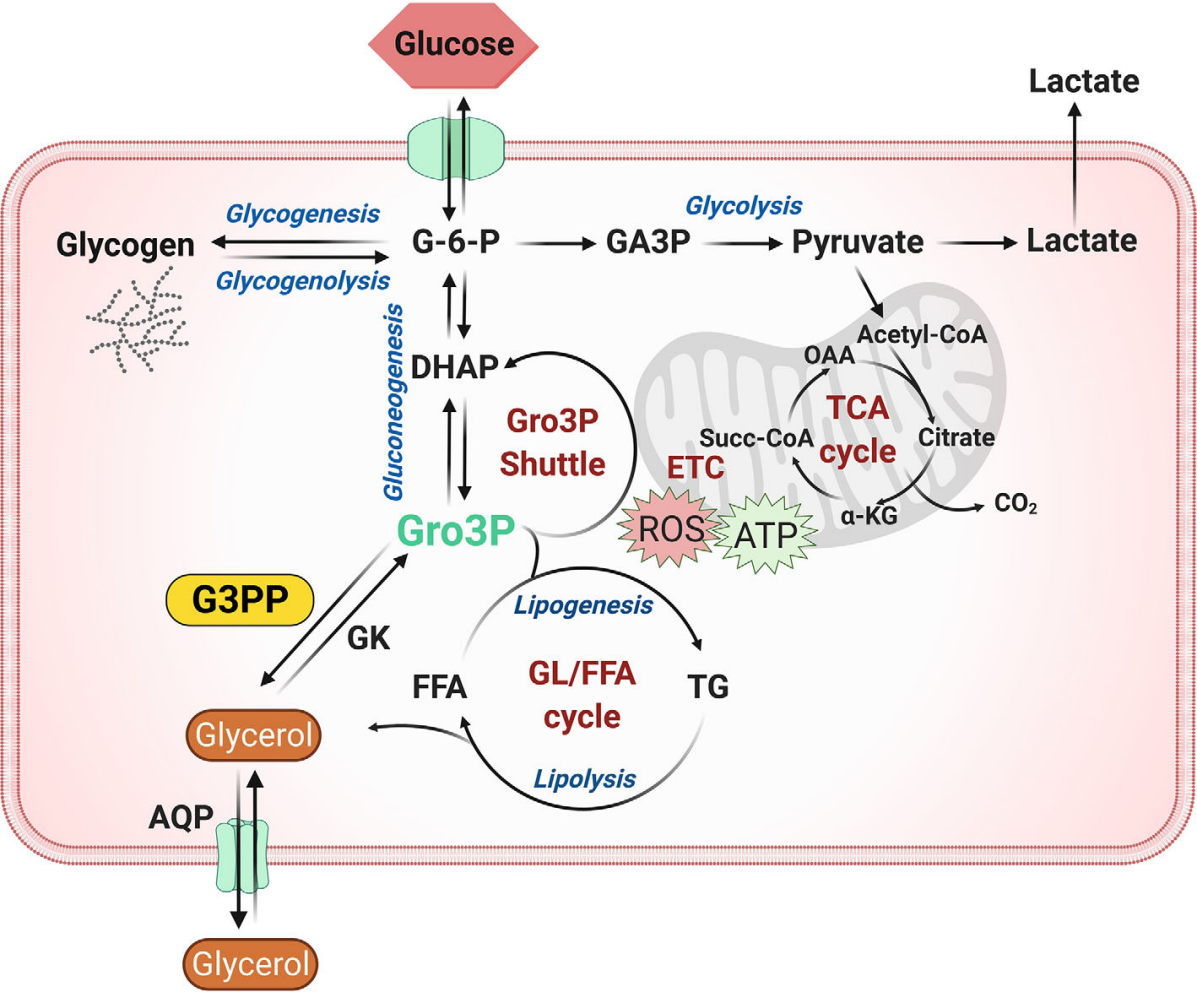
Role of G3PP in intermediary metabolism. Glycerol-3-phosphate (Gro3P) is a central metabolite at the intersection of four important pathways in most cells: 1) glycolysis; 2) glycerolipid synthesis and the glycerolipid/free fatty acid (GL/FFA) cycle; 3) gluconeogenesis (liver and kidney) and 4) energy metabolism *via* electron transfer shuttle to mitochondria. Gro3P can be produced from glucose *via* glycolysis or from lipolysis-derived glycerol by glycerol kinase. The cellular levels and availability of Gro3P are regulated by Gro3P phosphatase (G3PP), which dephosphorylates Gro3P to form glycerol. Under conditions of excess glucose supply, a buildup of Gro3P in the cell may cause an overflow of glucose carbons into glycolysis, lipid synthesis and electron transfer, leading to accumulation of fat and also elevated production of reactive oxygen species (ROS) in mitochondria. G3PP may act as a detoxification enzyme to protect the cells from glucotoxicity, excess fat synthesis and storage and oxidative damage by hydrolyzing Gro3P to glycerol, a less harmful molecule, that exits the cell through aquaglyceroporins. α-KG, α-ketoglutarate; AQP, aquaglyceroporin; DHAP, dihydroxyacetone phosphate; ETC, electron transport chain; FFA, free fatty acid; G-6-P, glucose-6-phosphate; G3PP, glycerol-3-phosphate phosphatase; GA3P, glyceraldehyde-3-phosphate; GK, glycerol kinase; GL/FFA cycle, glycerolipid/free fatty acid cycle; Gro3P, glycerol-3-phosphate; OAA, oxaloacetate; ROS, reactive oxygen species; Succ-CoA, succinyl-CoA; TCA cycle, tricarboxylic acid cycle; TG, triglycerides.

Glycolytically derived Gro3P is at the crossroads of glucose, lipid and energy metabolism in all cells, as it is the starting substrate for glycerolipid synthesis and also participates in the electron shuttle to transfer cytosolic reducing equivalents to mitochondrial electron transport chain for ATP synthesis (22, 24). Hydrolytic control of Gro3P in the cells by G3PP adds another level of metabolic regulation in animal cells that was not recognized previously, as the existence of G3PP is only recently established in mammalian cells. Significant evidence accumulated in the last five years suggests an important role for G3PP/PGP in the regulation of glucose and lipid metabolism in pancreatic islets, hepatocytes and adipocytes (**Figure 1**), which is summarized below.

### Pancreatic Islets

Cellular levels of Gro3P in INS-1(832/13) β-cells (35, 36) under physiological but elevated glucose concentrations (~10-16 mM) are sufficient to serve as the substrate for G3PP catalysis. In most cells, dihydroxyacetone-3-phosphate (DHAP) formed during glycolysis is partly converted to Gro3P. Pancreatic β-cells are not equipped to phosphorylate glycerol to Gro3P, as these cells express low levels of glycerol kinase (18, 37, 38). Modulation of cellular Gro3P levels by altering G3PP expression can impact the associated metabolic pathways. Thus, suppression of G3PP expression in rat pancreatic islets and INS-1(832/13) β-cells was found to lower glycerol production from glucose, in association with marked elevation of Gro3P, glycerolipid synthesis, glycolysis and glucose driven respiration, whereas overexpression of human G3PP in these cells produced opposite changes (22). In agreement with its anticipated role in the Gro3P shuttle to transfer electrons from the cytosol to mitochondria, the expression level of G3PP/PGP in rat pancreatic islets is inversely related to glucose-driven ATP synthesis. Interestingly, all the listed effects were apparent at elevated glucose (10-16 mM) with minimal changes at low (2-4 mM glucose). Thus, under elevated glucose concentration conditions, G3PP is able to control both glucose and lipid metabolism in β-cells (22).

### Hepatocytes

As in the case of pancreatic β-cells, Gro3P level in isolated rat hepatocytes (22) incubated at 5 or 25 mM glucose concentration was found to be sufficient to serve as substrate for G3PP. In hepatocytes, Gro3P can be formed from DHAP during glycolysis or by the phosphorylation of glycerol by glycerol kinase expressed in these cells (4). Similar to what was noticed in β-cells, suppression of G3PP expression in rat hepatocytes decreased glycerol production from glucose, increased intracellular Gro3P, glycerolipids, glycolysis, glucose driven respiration, and gluconeogenesis, whereas opposite changes were seen with overexpression of human G3PP in these cells (22). Interestingly, increased G3PP activity in hepatocytes also led to elevated fatty acid β-oxidation even at high glucose concentrations, which normally lower fatty acid oxidation (22). Importantly and as noted in ß-cells, these effects following changes in the expression levels of G3PP were prominent at high (25 mM) but less marked at low (5 mM) physiological basal glucose.

### Adipocytes

Adipocytes, from both white and brown adipose depots, synthesize and store large amounts of triglycerides and hydrolyze the same to release glycerol and FFA in response to hormonal and other cues. It is generally accepted that most of the glycerol released from adipocytes is of lipolytic origin. White adipocytes are known to conduct anaerobic glycolysis and produce lactate in large amounts from glucose (39, 40). It has recently been shown that in the presence of either glucose or fructose, mature differentiated 3T3-L1 adipocytes release copious amounts of lactate and glycerol, that cannot be accounted for their triglyceride stores and lipolysis, suggesting the presence of a mechanism for glycerol production from Gro3P, even though an enzyme for such reaction was not identified in adipocytes (41). In the later studies, these authors showed that G3PP/PGP is expressed in the primary rat adipocytes and contributes to glycerol production and that its expression is much higher than that of glycerol kinase, thereby ensuring removal of glycerol produced from Gro3P *via* aquaporin-7 in these cells (42). The same group studied glycerol metabolism at various glucose concentrations (3, 5, 7, or 14 mM) with and without insulin. When medium glucose was at basal level, most glycerol came from lipolysis, but when glucose was high, the release of glycerol *via* breakup of Gro3P was predominant (43). Under conditions of excess glucose supply, adipocytes take-up glucose and metabolize *via* glycolysis to Gro3P. But if not enough FFA is simultaneously available, the produced Gro3P is hydrolyzed to export glycerol *via* aquaporin-7. Adipocytes do not have a significant level of *de novo* lipogenesis machinery (44) unlike hepatocytes, so they cannot generate acyl-CoA (*de novo*) to esterify Gro3P to triglyceride. It is necessary to hydrolyze Gro3P, as it can be toxic when present in excess and generate toxic reactive oxygen species or even inhibit glycolysis, unless removed as glycerol. Thus, G3PP appears to play an important role in regulating glycolysis and glycerolipid metabolism in adipocytes at elevated glucose levels primarily (42, 45).

### Evidence From Embryonic Cells With Inactive Mutant G3PP

Regulation of glycerolipid metabolism by G3PP/PGP has recently been shown to be essential during development. Thus, in E8.5 mouse embryos expressing catalytically inactive G3PP/PGP (*PGP^D34N/D34N^*) elevated diacylglycerols and triglycerides were noticed, indicating increased lipogenesis from accumulating Gro3P in these knock-in embryos (46). As the *PGP^D34N/D34N^* knock-in mouse embryos do not survive beyond E11.5, it appears that G3PP/PGP is essential during development, possibly due to its role in glucose and glycerolipid metabolism (46).

Overall the data indicate that G3PP is a glucose concentration-dependent enzyme at the nexus of glucose and lipid metabolism that plays a role in glucose, energy and lipid metabolism primarily at elevated concentrations of glucose and cellular Gro3P.

## Physiological Roles of G3PP/PGP

As G3PP/PGP plays a role in glucose, lipid, and energy metabolism (**Figure 1**), this enzyme is likely implicated in various physiological and pathological processes related to nutrient excess. It was earlier suggested that G3PP/PGP plays a role in the removal of 2-phosphoglycolate generated during DNA repair processes (46), but this is questionable as in mammalian cells, 2-phosphoglycolate levels are either very low under normal conditions (22) or below detectable levels, even under stress conditions (46, 47). Similarly, the 2-phosphoglycolate hydrolytic ability of G3PP/PGP has been implicated (48) in the control of the bifunctional glycolytic enzyme 1,3-diphosphoglycerate mutase/2,3-diphosphoglycerate (DPG) phosphatase, which regulates hemoglobin binding to oxygen in the RBC. DPG phosphatase hydrolyzes 2,3-diphosphoglycerate, which is known to lower the affinity of hemoglobin for oxygen and thus promote the release of oxygen from oxyhemoglobin (49). The proposed role for PGP in RBC in this process is to control the levels of 2-phosphoglycolate, which potently activates the DPG phosphatase (48). However, the concentration of 2-phosphoglycolate in RBC (~4 µM) is nearly 200 fold less than its Km for G3PP/PGP (26), and at such low concentration, 2-phosphoglycolate may not be available as a substrate for G3PP/PGP in RBC (50), questioning this role of G3PP/PGP in RBC.

### Detoxification of Metabolic By-Products of Enzymatic Reactions

Toxic metabolic side products are generated in cells due to mutations in enzymes, changes in metabolic flux or due to the enzyme reaction with alternative substrates at low rates as the specificity of many enzymes is not absolute. Thus, it was shown that GAPDH can also catalyze the conversion of the pentose phosphate pathway metabolite erythrose-4-phosphate to 4-phosphoerythronate (51), though at a much lower rate (32). Similarly, pyruvate kinase was shown to phosphorylate lactate to 2-phospholactate (52). 4-Phosphoerythronate and 2-phospholactate were shown to inhibit 6-phosphogluconate dehydrogenase and phosphofructokinase-2, respectively (32). As 4-phosphoerythronate and 2-phospholactate accumulate only in *PGP* deleted HCT116 cells (32) and malarial parasite Plasmodium (33, 53), it was suggested that G3PP/PGP can act like a ‘metabolic repair enzyme’ and hydrolyze these two toxic metabolites. However, as the concentrations of these metabolites in wild type cells with normal expression of G3PP/PGP are much lower than their corresponding Km for purified mouse G3PP/PGP, it is plausible that only under stress conditions when these metabolites accumulate significantly, G3PP/PGP may hydrolyze them and act as a detoxification enzyme of various phospho-metabolites, besides its action on Gro3P.

### Adipose Tissue and Liver Metabolism

Increased adiposity leads to obesity and is an important risk factor for type 2 diabetes (T2D) and other cardiometabolic diseases. Synthesis and storage of triglycerides is critical in the expansion of adipose tissue, and recent studies demonstrated a role for G3PP/PGP in the control of lipogenesis by the hydrolysis of Gro3P in primary white adipocytes (42). We reported that the expression level of G3PP in mouse visceral, subcutaneous and brown fat is nutritionally regulated (fed *vs*. fasting state and low *vs*. high-fat diet (20). The precise role of G3PP in various adipose depots and whole-body energy homeostasis remains to be defined.

Liver plays key role in the synthesis and secretion of lipoproteins and gluconeogenesis and these processes are dependent on the availability of Gro3P in hepatocytes. We reported that in isolated rat primary hepatocytes incubated at elevated glucose (25 mM), the levels of Gro3P and glycolysis as measured by lactate release and glycerolipid synthesis as indicated by diacylglycerol and triglyceride levels, were all higher when G3PP/PGP expression was suppressed using RNAi. Opposite effects were noted in hepatocytes with human G3PP overexpression (22). All these effects due to variations in the expression level of G3PP were glucose concentration dependent as they were much less at low 5 mM glucose. Interestingly, the toxic metabolite 2-phosphoglycolate was found at very low levels in hepatocytes but was measurable in these cells at 5 and 25 mM glucose, but its concentration did not change upon RNAi-suppression of G3PP, indicating that G3PP has little or no role in modulating 2-phosphoglycolate levels under normal conditions. In addition, we noticed that adenoviral vector mediated overexpression of G3PP in rat liver, led to decreased gluconeogenesis from glycerol and also enhanced secretion of high density lipoprotein, *in vivo* (22). Thus, G3PP/PGP in adipocytes and hepatocytes likely has a significant role in the regulation of their physiological functions.

### Influence of Nutrients, Hormones and Dietary State

Considering that G3PP/PGP is an important metabolic enzyme that regulates glucose and lipid metabolism and nutri-stress, expression of this enzyme is likely regulated by multiple factors including nutritional status. Thus, we observed that overnight fasting in mice lowered G3PP expression at mRNA and protein levels in brown adipose tissue while its expression increased in visceral white adipose tissue (22). It was suggested that such depot specific inverse change in G3PP expression ensures supply of lipolysis-derived glycerol upon fasting, from white adipose to liver and kidney for gluconeogenesis, rather than being used for re-esterification. However, feeding of 60% high fat diet to mice led to elevated G3PP expression in brown adipose but decreased in white adipose depots, so that Gro3P is made available for the esterification of excess FFA for storage as triglycerides. High fat diet increased G3PP mRNA expression in heart and also testis also, probably as a defense mechanism to prevent build-up of fat in these organs, which otherwise may have pathological consequences (22). Expression of G3PP was found to be unaltered by increasing glucose concentration (7 to 14 mM) in differentiated 3T3-L1 adipocytes (42) and also in primary visceral white adipocytes from rats (45) and exogenous insulin was also without any effect on G3PP expression at various glucose concentrations (43). Conversely, the expression of three G3PP/PGP homologues (K09H11.7; C53A3.2; F44E7.2) in *C. elegans* was found to be upregulated upon exposure to high glucose concentration, accompanied by elevated glycerol production (54). Interestingly, it was described that G3PP mRNA expression in female rat perigonadal white adipose tissue is much higher than in males, but if this corresponds with elevated G3PP activity in female adipose tissue is not known (45). It is yet to be determined if sex hormones influence the expression of G3PP in any tissue, even though testis as a whole organ is found to have the most expression of G3PP among various body tissues (22). Thus, the expression of G3PP appears to be controlled by nutritional status in a tissue and organism dependent manner, which adds another level of regulation of energy metabolism, and further work is needed to fully understand the hormonal regulation of this enzyme’s expression.

### Insulin Secretion

Insulin secretion in pancreatic β-cells is driven by the intracellular metabolism of glucose and other fuels, in particular by the so-called metabolic coupling factors derived from glycolysis, mitochondrial and lipid metabolism, such as the ATP/ADP ratio and monoacylglycerol (38). Our earlier studies showed that changes in the expression level of G3PP/PGP in β-cells alters glucose and lipid metabolism and the glycerolipid/FFA cycle in ß-cells, which are known to produce coupling factors for glucose induced insulin secretion. Thus, downregulation of G3PP in INS1(832/13) β-cells and rat islets was found to enhance GSIS in association with elevated production of ATP and glycerolipids, such as diacylglycerols, which are known to act as signals to promote insulin secretion, while G3PP over-expression led to slowed down GSIS response accompanied by reduced ATP and glycerolipid production (22). Thus, G3PP/PGP is a new player in ß-cell metabolic signaling and insulin secretion.

## G3PP in Human Diseases

Considering that Gro3P occupies a central position in glucose, lipid and energy metabolism, enzymes that generate and use this metabolite are likely to have important regulatory roles and any defects in these enzymes can have pathological consequences. Thus, glycerol kinase deficiency in humans is an X-chromosome linked disease and is reported to be associated with abnormalities in lipid metabolism, susceptibility to diabetes, metabolic acidosis, etc. (55). Glycerol kinase knockout mice were found to have severely disturbed fat metabolism, hyperglycerolemia and postnatal growth retardation and death by 3-4 days of age (56). Mitochondrial glycerol-3-phosphate dehydrogenase deletion in livers was found to lead to hepatic steatosis due to enhanced lipogenesis in mice (57) and a rare case of genetic deficiency of this enzyme was found to be associated with mental retardation (58). In addition, cytosolic glycerol-3-phosphate dehydrogenase KO mice were found to have elevated body weight, increased compensatory gluconeogenesis from alanine, increased fatty acid oxidation in skeletal muscle, but reduced gluconeogenesis from glycerol (59). Mutations in cytosolic glycerol-3-phosphate dehydrogenase were shown to be associated with transient hypertriglyceridemia in children (60). Similarly, Gro3P acyltransferases, which esterify Gro3P to lysophosphatidic acid, were implicated in obesity and hepatic steatosis and insulin resistance (61). Collectively, it appears that all the enzymes that are directly involved in the synthesis and utilization of Gro3P play critical roles in the whole body metabolism and their compromised activity can have pathological consequences.

Despite the fact that *PGP* gene is essential for mammalian embryogenesis and development (46), there are no studies showing a direct “cause and effect” relationship between G3PP/PGP and the pathogenesis of human diseases. However, considering the role of G3PP/PGP in the regulation of glucose, lipid and energy metabolism, it may have a protective function against cardiometabolic diseases due to excess nutrient fuel supply.

### Nutri-Stress and Glucolipotoxicity

Excess supply of nutrient fuels is the primary cause of cellular dysfunction in various organs, including pancreatic islets, heart muscle and liver, that eventually causes cardiometabolic diseases (62, 63), and we have recently termed this as ‘nutri-stress’ (64). Indeed, toxicity manifested as apoptosis in tissues due to the combined presence of excess glucose and fatty acids is called glucolipotoxicity (65–67). We have proposed that the diversion of glucose carbons to glycerol is deployed as a defense mechanism by pancreatic β-cells to evade toxic effects of excess glucose, as β-cells cannot re-use glycerol, which leaves the cell (35). Thus, the expression level of G3PP, which is responsible for the direct conversion of glucose carbons to glycerol, was found to be inversely related to glucotoxicity and glucolipotoxicity in β-cells (22). Elevated G3PP/PGP expression in β-cells has also been shown to slow-down the β-cell response to secrete increased levels of insulin in the presence of high concentrations of glucose (22), an effect that is anticipated to prevent hyperinsulinemia as well as β-cell exhaustion and dysfunction. Thus, the emerging view is that G3PP/PGP plays an important role in the β-cells not only to regulate glucose and lipid metabolism but also as a glucose excess security valve to alleviate metabolic stress in these cells and to prevent hyperinsulinemia, which leads to insulin resistance, obesity and T2D in the face of excess nutrient supply. In addition to β-cells, G3PP/PGP expression level is also relevant in preventing excess synthesis and storage of fat in the liver and thus hepatic steatosis, and also in slowing down hepatic glucose production, a significant problem in T2D (22).

### Cardiometabolic Disorders

Chronic nutri-stress is the root cause of cardiometabolic disorders such as metabolic syndrome, T2D, obesity, atherosclerosis, and non-alcoholic fatty liver disease (64, 68). Nutri-stress promotes lipogenesis with associated accumulation of fat in adipose tissue and other tissues (18, 35, 37), oxidative damage due to excessive ROS production *via* mitochondrial metabolism and leads to aggravated local and systemic inflammation (69). The increased cellular level of Gro3P derived from glucose metabolism is likely at the center of many of these pathogenic pathways. Therefore, curtailing the excess buildup of Gro3P by G3PP likely alleviates the metabolic stress and fuel surfeit toxicity to the cells. Thus, *in vitro* and *in vivo* studies in rats with G3PP overexpression suggested several beneficial effects of elevated G3PP activity in the liver and pancreatic islets against metabolic complications due to excess nutrients (22).

## Conclusions and Perspective

Despite the earlier belief that mammals do not possess a G3PP like enzyme, there is an overwhelming evidence now, as summarized in this review, that indeed such an enzyme exists in mammalian cells and plays a major role in the regulation of metabolic and physiological processes, disturbances of which could lead to cardiometabolic diseases. Activity of G3PP in pancreatic β-cells appears to be an important player in the regulation of GSIS and also in preventing nutri-stress. In other cell types, including hepatocytes and adipocytes, G3PP seems to control glucose and lipid metabolism and excessive fat buildup. There are still several knowledge gaps concerning the role of G3PP in other organs, including muscle and heart, whose functions are affected in cardiometabolic diseases. In addition, the proposed role of this enzyme as a metabolic repair enzyme needs to be further studied for its relevance in mammalian cells under normal physiological conditions. Several of these knowledge gaps can be addressed using appropriate animal models, including tissue-specific knockout and graded overexpression models. More detailed genetic studies are also needed focusing on the association of the *PGP* gene and its variations with cardiometabolic diseases. Considering that elevated activity of G3PP protects against nutri-stress, excess fat buildup, hyperinsulinemia, hepatic glucose production and fatty liver disease, this enzyme can be a potential therapeutic target for cardiometabolic diseases.

## Author Contributions

EP and AA-M contributed equally to this review. MP and SM conceptualized the topic and the outline for the review. EP, AA-M, and M-LP collected literature information and compiled it. EP and MP prepared the Figure. SM and MP wrote the review and edited it. RA and FA-M contributed by analytical reading of the review and valuable suggestions to improve. All authors contributed to the article and approved the submitted version.

## Funding

This study was supported by funds from the Canadian Institutes of Health Research (to MP and SM) and Montreal Medical International/Dasman Diabetes Institute (to MP, SM, and RA). EP was supported by a postdoctoral fellowship from the Canadian Diabetes Association.

## Conflict of Interest

The authors declare that the research was conducted in the absence of any commercial or financial relationships that could be construed as a potential conflict of interest.
